# Identification of Reduced Host Transcriptomic Signatures for Tuberculosis Disease and Digital PCR-Based Validation and Quantification

**DOI:** 10.3389/fimmu.2021.637164

**Published:** 2021-03-02

**Authors:** Harriet D. Gliddon, Myrsini Kaforou, Mary Alikian, Dominic Habgood-Coote, Chenxi Zhou, Tolu Oni, Suzanne T. Anderson, Andrew J. Brent, Amelia C. Crampin, Brian Eley, Robert Heyderman, Florian Kern, Paul R. Langford, Tom H. M. Ottenhoff, Martin L. Hibberd, Neil French, Victoria J. Wright, Hazel M. Dockrell, Lachlan J. Coin, Robert J. Wilkinson, Michael Levin

**Affiliations:** ^1^Section of Paediatrics, Department of Infectious Disease, Faculty of Medicine, Imperial College London, London, United Kingdom; ^2^National Public Health Speciality Training Programme, South West, United Kingdom; ^3^Imperial Molecular Pathology, Imperial Healthcare Trust, Hammersmith Hospital, London, United Kingdom; ^4^Centre for Haematology, Faculty of Medicine, Imperial College London, London, United Kingdom; ^5^Institute for Molecular Bioscience, University of Queensland, Brisbane, QLD, Australia; ^6^School of Public Health and Family Medicine, Faculty of Health Sciences, University of Cape Town, Cape Town, South Africa; ^7^Brighton and Sussex Medical School, Brighton, United Kingdom; ^8^Brighton and Malawi Liverpool Wellcome Trust Unit, Blantyre, Malawi; ^9^Nuffield Department of Medicine, University of Oxford, Oxford, United Kingdom; ^10^Oxford University Hospitals National Health Service (NHS) Foundation Trust, Oxford, United Kingdom; ^11^Malawi Epidemiology and Intervention Research Unit, Chilumba, Malawi; ^12^London School of Hygiene & Tropical Medicine, London, United Kingdom; ^13^Karonga Prevention Study, Chilumba, Malawi; ^14^Paediatric Infectious Diseases Unit, Red Cross War Memorial Children's Hospital, Cape Town, South Africa; ^15^Department of Paediatrics and Child Health, University of Cape Town, Cape Town, South Africa; ^16^Division of Infection and Immunity, Faculty of Medical Sciences, University College London, London, United Kingdom; ^17^Brighton and Sussex Medical School, University of Sussex, Brighton, United Kingdom; ^18^Brighton and Sussex University Hospitals National Health Service (NHS) Trust, Brighton, United Kingdom; ^19^Department of Infectious Diseases, Leiden University Medical Center, Leiden, Netherlands; ^20^Faculty of Infectious and Tropical Diseases, London School of Hygiene and Tropical Medicine, London, United Kingdom; ^21^Tropical and Infectious Disease Unit, Royal Liverpool and Broadgreen University Hospitals National Health Service (NHS) Trust, Liverpool, United Kingdom; ^22^Centre for Global Vaccine Research, Institute of Infection & Global Health, University of Liverpool, Liverpool, United Kingdom; ^23^Department of Immunology and Infection, and Tuberculosis (TB) Centre, London School of Hygiene and Tropical Medicine, London, United Kingdom; ^24^The Francis Crick Institute, London, United Kingdom; ^25^Department of Medicine, Imperial College London, London, United Kingdom; ^26^Wellcome Centre for Infectious Diseases Research in Africa, Institute of Infectious Diseases and Molecular Medicine, University of Cape Town, Cape Town, South Africa

**Keywords:** tuberculosis, transcriptomics, dPCR, gene expression, signatures, biomarkers

## Abstract

Recently, host whole blood gene expression signatures have been identified for diagnosis of tuberculosis (TB). Absolute quantification of the concentrations of signature transcripts in blood have not been reported, but would facilitate diagnostic test development. To identify minimal transcript signatures, we applied a transcript selection procedure to microarray data from African adults comprising 536 patients with TB, other diseases (OD) and latent TB (LTBI), divided into training and test sets. Signatures were further investigated using reverse transcriptase (RT)—digital PCR (dPCR). A four-transcript signature (*GBP6, TMCC1, PRDM1*, and *ARG1*) measured using RT-dPCR distinguished TB patients from those with OD (area under the curve (AUC) 93.8% (CI_95%_ 82.2–100%). A three-transcript signature (*FCGR1A, ZNF296, and C1QB*) differentiated TB from LTBI (AUC 97.3%, CI_95%_: 93.3–100%), regardless of HIV. These signatures have been validated across platforms and across samples offering strong, quantitative support for their use as diagnostic biomarkers for TB.

## Introduction

Despite over a century's research effort to identify new diagnostic tools we still lack diagnostic tests for tuberculosis (TB) that are sensitive, affordable and robust. The majority of TB diagnostics are based on identifying the pathogen in sputum, by microscopy, culture or PCR. However, current methods fail to identify the pathogen in a significant proportion of cases, either due to inadequacies in sputum collection, paucibacillary disease, HIV infection or in patients with extrapulmonary forms (1). As a result the World Health Organization (WHO) estimates that approximately three in every ten TB cases go unreported or undiagnosed (2). Given the problems associated with using sputum as a clinical sample, the WHO and the Foundation for Innovative New Diagnostics published a target product profile (TPP) for a non-sputum biomarker test in 2014 (3). This specified the seven proposed key characteristics of a rapid biomarker-based non-sputum-based test for detecting TB including minimal and optimal sensitivity and specificity of such a test and also discussed sample accessibility, time to result, maintenance and cost.

Recent years have seen a rise in the emergence of host-response-based infectious disease diagnostics. These detect evidence of a host immune response to an infection, which is advantageous when there are very low numbers of the pathogen in the body or when pathogens colonize inaccessible sites. A number of disease specific “omic” signatures have been identified, facilitated by advances in technology to analyse the genome, transcriptome, epigenome, lipidome, metabolome, and proteome in a high-throughput and quantitative manner (4). As well as improving our understanding of the pathogenesis of a range of infectious diseases, these signatures have the potential to be used as diagnostic biomarkers.

Gene expression studies have significantly enhanced our knowledge of the roles of various components of the immune system in TB disease (5–7). A number of gene expression signatures have been published that can distinguish TB from healthy controls (HCs) and correlate with disease progression (8–10). These could serve as important indicators of disease progression from latent TB infection (LTBI) to TB, and therefore guide antibiotic selection (11).

The most clinically important need is for biomarkers to distinguish TB from the range of other conditions with similar clinical presentation. TB shares symptoms and clinical signs with many other diseases (OD), including a wide range of infectious, inflammatory and malignant conditions, such as pneumonia or other HIV-associated opportunistic infections. Distinguishing between TB and OD is particularly important in patients living with HIV, because extrapulmonary TB is more common in these patients (12, 13) such that most sputum-based tests are poorly sensitive, and HIV-associated malignancies or opportunistic infections can have similar clinical presentations. However, the majority of TB gene expression studies published to date have compared TB cohorts to HCs, LTBI or patients with OD, mostly in the absence of HIV infection.

A previous study Kaforou et al. (14) addressed these issues by studying patients with symptoms suggestive of TB in Malawi and South Africa (including both HIV-infected and uninfected persons) and classifying them as TB, LTBI or OD. Blood gene expression signatures were identified using genome-wide microarrays that distinguished TB from OD and LTBI (14). A 44-transcript signature was found to distinguish TB from OD with sensitivity of 93% (CI_95%_ 83–100) and specificity of 88% (CI_95%_ 74–97). A 27-transcript signature distinguished TB from LTBI with sensitivity of 95% (CI_95%_ 87–100) and specificity of 90% (CI_95%_ 80–97). These signatures showed only slightly reduced accuracy in HIV-coinfected individuals (14).

Further reduction in the number of transcripts comprising these gene expression signatures makes their use as diagnostic markers more feasible for clinical translation, particularly at the point-of-care and in resource-limited settings (15). This has been the subject of significant research effort and a number of bioinformatics approaches have been employed. Sweeney et al. identified a three-gene signature for TB, comprised of *GBP5, DUSP3*, and *KLF2* in a meta-analysis of publicly available gene expression microarray data (16). Maertzdorf et al. used random forest models and confidence interval decision trees to identify a four-transcript signature comprising *GBP1, IFITM3, P2RY14*, and *ID3*, that distinguished between TB and HC, regardless of HIV infection status (17). Other recent studies identified minimal gene expression signatures in populations from high-endemic countries that predict progression from latent infection to active TB disease with accuracy, excluding cases with HIV co-infection (18, 19).

Quantification of individual TB gene expression signature transcripts would be useful to determine the limits of detection required for diagnostic tests based on these signatures. The established method of choice for performing absolute quantification of nucleic acids is quantitative PCR (qPCR), where amplicon generation is measured in real time and related back to the starting concentration of template. While RNA-seq has emerged as a powerful technique for investigating RNA species within a given sample, it can only provide *relative* quantification of RNA species (20). In recent years, digital PCR (dPCR) has emerged as a promising alternative to qPCR. dPCR is a useful method for quickly and efficiently providing absolute quantification of individual mRNA species and has been shown to be more reproducible and less prone to inhibition than qPCR (21, 22). The high precision offered by dPCR makes it ideally suited to the detection of rare point mutations and the accurate detection of low microbial loads, among other applications (23–25).

We hypothesized that we could further reduce the number of transcripts comprising the previously reported signatures distinguishing TB from OD and LTBI Kaforou et al. (14) using feature selection algorithms applied to microarray data, and that reverse transcription-dPCR (RT-dPCR) could be used to quantify the concentrations of individual gene transcripts in purified RNA from whole blood. We postulated that this cross-sample, cross-platform (microarray and RT-dPCR), cross-population study will aid the advance of the TB transcriptomics field toward developing and establishing the use of host transcriptomics for TB diagnosis.

## Materials and Methods

### Ethics Statement

The study was approved by the Human Research Ethics Committee of the University of Cape Town, South Africa (HREC012/2007), the National Health Sciences Research Committee, Malawi (NHSRC/447), and the Ethics Committee of the London School of Hygiene and Tropical Medicine (5212). Written information was provided by trained local health workers in local languages and all patients provided written consent.

### Derivation of Reduced Signatures Using Microarray Data

The patient cohorts recruited in South Africa and Malawi for the original prospective cohort microarray study were fully described previously, including the diagnostic procedures and patient assignment as TB, OD or LTBI (14). In addition, the whole-blood genome-wide expression measured in this cohort was reported (14), and made publicly available at NCBI's Gene Expression Omnibus, accessible through GEO Series accession number GSE37250. The microarray data was pre-processed as described in (14). Data from the processed and normalized expression set were split randomly into training and test set (80–20 split). FS-PLS (26, 27) was employed in order to generate smaller gene expression signatures. FS-PLS is an iterative forward selection algorithm which at each step selects the most strongly associated variable after projecting the data matrix into a space orthogonal to all the variables previously selected. It combines the dimensionality reduction strength of PLS and the model simplicity and interpretability of FS regression. The classificatory performance of the signatures was evaluated in the test set using the disease risk score method (DRS), as in (14). The derived signatures were further validated in two publicly available gene expression studies (5, 28) (Supplementary Material). The FS-PLS code is available for download and use (27).

### Power Calculations for RT-dPCR Study Size

For the retrospective RT-dPCR study, as the discrimination using the DRS had a binary outcome and followed a binomial distribution, in order to achieve a statistic significance level of 0.05, and assuming the dPCR sensitivity to be at least 75% for patient classification, we used 40 samples for each comparison (TB vs. OD and TB vs. LTBI) to assess the performance of each signature, with equal numbers of samples for each group (n_TB_ = 20, n_OD_ = 20, n_LTBI_ = 20) (Supplementary Tables 1, 2). Samples were chosen at random from a microarray test patient cohort for TB vs. OD, stratified for HIV status and country of origin, which had not been used to derive the signature. An additional 10 LTBI HIV-infected and 10 LTBI HIV-uninfected samples from the test microarray cohort were analyzed.

### Patient Characteristics for RNA Samples Used in the RT-dPCR

Patient recruitment was conducted in two highly contrasting study sites in Cape Town, South Africa and Karonga District, Northern Malawi. Patients were classified as having active TB disease only upon culture confirmation. Patients were deemed to have OD if they presented with symptoms that might suggest the possibility of TB disease, but for whom an alternative diagnosis was found and TB treatment was not administered. These patients were followed up 26 weeks post diagnosis to confirm they remained TB-free. Healthy LTBI controls were classified according to the results of interferon-gamma release assay (IGRA) and tuberculin skin test (TST) investigations (14).

### RNA Purification From Whole Blood and Storage

2.5 ml whole blood was collected at the time of recruitment (before or within 24 h of commencing TB treatment in suspected patients) in PAXgene blood RNA tubes (PreAnalytiX), frozen within 3 h of collection, and later extracted using PAXgene blood RNA kits (PreAnalytiX). RNA was shipped frozen and stored at −80°C.

### Assessment of RNA Purity and Integrity

Before proceeding with reverse transcription, the RNA quality of the samples was assessed using an Agilent 2100 Bioanalyzer (Agilent Technologies, Santa Clara, CA, USA).

### Reverse Transcription of Purified RNA From Whole Blood

RNA concentration was measured using a NanoDrop 2000c (Thermo Scientific) and 500 ng was used for the reverse transcription reaction in a total volume of 10 μL nuclease-free H_2_O. RT was performed in one batch using the High-Capacity cDNA RT Kit (Applied Biosystems) according to the manufacturer's instructions. The cycle was 25°C for 10 min, 37°C for 120 min, 85°C for 5 min, followed by a hold at 4°C. cDNA samples were stored at −20°C for fewer than 6 months before use.

### dPCR Using the QuantStudio^TM^ Platform

Up to 5 μL of RT product was added to 7.5 μL QuantStudio 3D Digital PCR Master Mix (Thermo Fisher Scientific), 0.75 μL of TaqMan Assay (20X) (Thermo Fisher Scientific) (see Supplementary Table 3) and the volume made up to 15 μL using nuclease-free H_2_O (Supplementary Figure 3). All TaqMan Assays were inventoried and none were custom-made. At least one no template control was used for each TaqMan assay on each PCR run. The reaction mix was applied to each QuantStudio 3D Digital PCR 20K Chip (Applied Biosystems) according to the manufacturer's instructions. The dPCR was run on a GeneAmp PCR System 9700 (Applied Biosystems) with a cycle of 10 min at 96°C, followed by 39 cycles of 60°C for 60 s and 98°C for 30 s, followed by 2 min at 60°C before holding at 10°C. Chips were read, and absolute quantification (copies per μL) determined using the QuantStudio 3D Digital PCR Instrument (Thermo Fisher Scientific).

### Data Analysis RT-dPCR

Data was exported and analyzed using QuantStudio 3D AnalysisSuite Cloud Software Version 3.0.3 (Thermo Fisher Scientific). The quantification algorithm selected was Poisson. The software assesses whether the data on a chip is reliable based upon loading, signal, and noise characteristics and displays quality indicators for each chip. Any chip that gave a precision value of >10% was deemed to have failed and was repeated. Similarly, if the negative and positive wells did not separate into distinct populations, the sample and probe combination was repeated. This failure to separate into two populations could be caused by the chips leaking, evaporation or a loading issue of the sample onto the chip. This methodology is further explained in the supporting information (Supplementary Figure 1) and all dilutions, FAM call thresholds and lambda values are given in Supplementary Table 4, in accordance with the MIQE guidelines (21). The output given by the QuantStudio software is in copies/μL. This value was then corrected according to the dilution of cDNA used for the dPCR in order to determine the absolute concentration of a given transcript in purified RNA samples (Supplementary Figure 3). RT-dPCR derived copies per μL values are reported. The DRS method was used to classify patients on the basis of log_2_ (copies per μL).

### Statistical Analysis

The datasets were analyzed in “R” Language and Environment for Statistical Computing version 3.4.1 (29, 30). In order to evaluate the performance of the DRS as a binary classifier, the area under the curve (AUC) for a receiver operating characteristic (ROC) curve was calculated, as well as the sensitivity and specificity using pROC (29). The calculation of the confidence intervals (CI) for the AUC was based on the DeLong method (31), an asymptotically exact method to evaluate the uncertainty of an AUC, except for the one case that AUC = 100%, where we used a smoothed ROC followed by DeLong for the calculation of the lower 95% bound. For each data set we report the point estimate for sensitivity as the closest value >90% (as specified in the WHO TPP) and the corresponding specificity.

## Results

### Discovery and Validation of Small Signatures From Microarray Data Using FS-PLS and DRS

**Figure 1:**
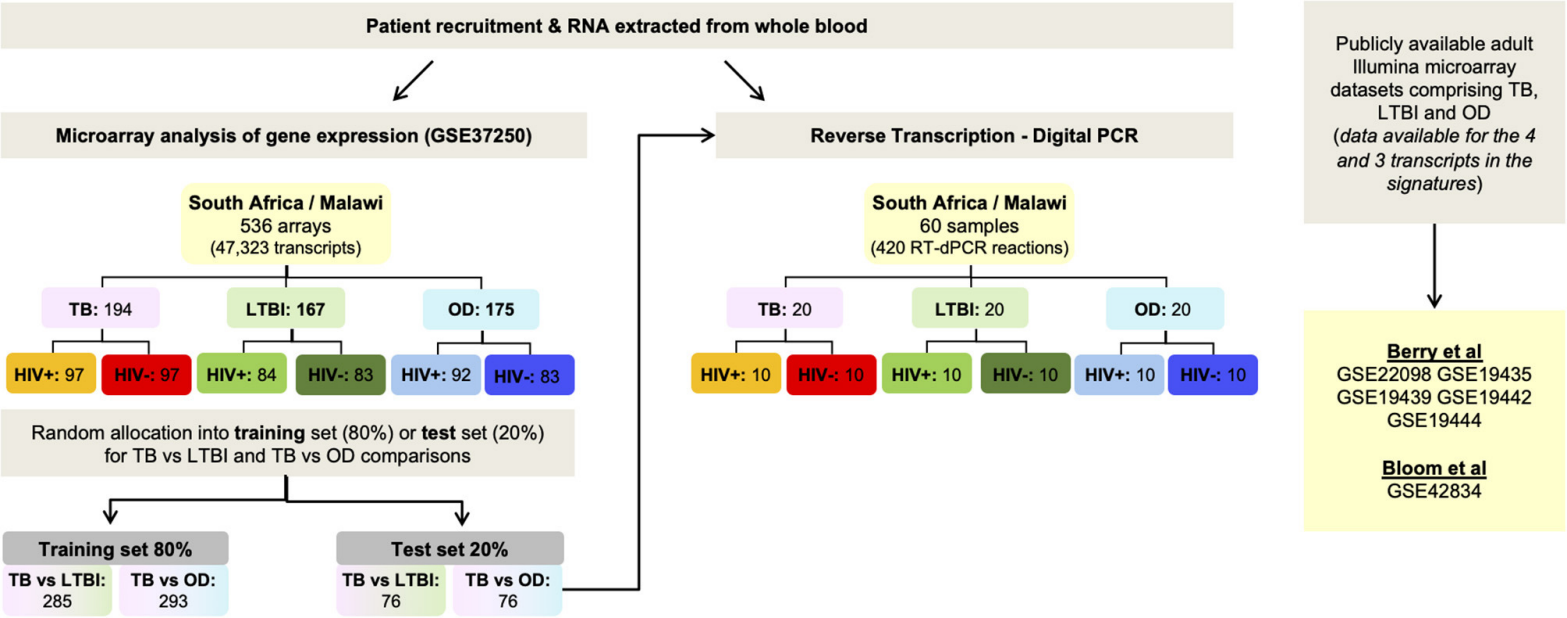
Workflow. Identification of small signatures for TB/LTBI and TB/OD from microarray data using Forward Selection-Partial Least Squares (FS-PLS), followed by classification performance in a separate test set and finally, validation using RT-dPCR using the test set. Performance of the signatures was also assessed in publicly-available microarray datasets. OD, other diseases; LTBI, latent TB infection.

**Figure 2:**
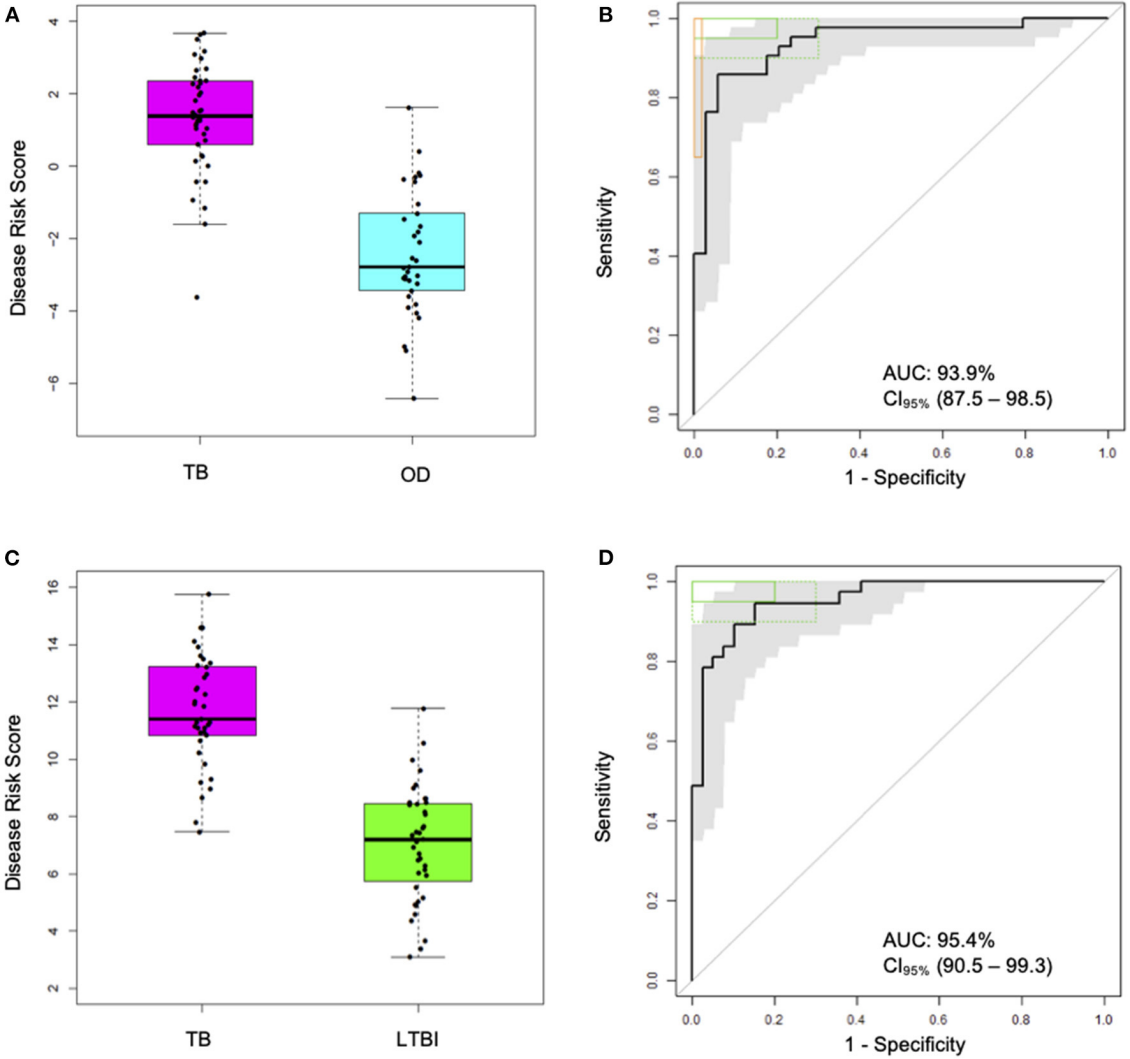
Classification performance of the FS-PLS-derived four-transcript signatures for TB/OD and three-transcript for TB/LTBI using microarray gene expression data (only test dataset shown). **(A)** Box plots of DRS and **(B)** receiver operating characteristic (ROC) curve based on the TB/OD FS-PLS signature applied to the combined HIV ± TB and OD SA/Malawi cohorts (TB DRS vs. OD DRS Mann–Whitney *p* = 1.33 × 10^−13^) **(C)** Box plots of DRS and **(D)** ROC curve based on the TB/LTBI FS-PLS signature applied to the combined HIV ± TB and LTBI SA/Malawi cohorts (TB DRS vs. LTBI DRS Mann–Whitney *p* = 4.92 × 10^−15^). Gray shaded areas represent the 95% CIs of the ROC curve sensitivities, plotted at 0.5% specificity intervals. ROC curves **(B,D)** have been benchmarked against target criteria for a tuberculosis triage test (green boxes): Minimum criteria (90% sensitivity, 70% specificity) are indicated by the dashed green boxes and optimum criteria (95% sensitivity, 80% specificity) are indicated by the green solid boxes. The TB vs. OD ROC curve **(B)** has been benchmarked against the minimum criteria for a confirmatory test (65% sensitivity, 98% specificity) which are indicated by the orange box. AUC, area under the curve; OD, other diseases; LTBI, latent TB infection.

In order to derive reduced gene expression signatures with diagnostic potential, the variable selection method, FS-PLS, was applied to the previously published microarray data (80% training set) (*n* = 293 for TB vs. OD, *n* = 285 for TB vs. LTBI including HIV co-infected cases), tested in the test set (*n* = 76 TB vs. OD and TB vs. LTBI including HIV co-infected cases), and further validated in two other publicly available studies (Figure 1) (5, 28). Using the FSPLS method we identified a signature comprising four transcripts for TB/OD in the training set and a signature comprising three transcripts for TB/LTBI; the signatures are detailed in Tables 1A,B, respectively. The TB/OD FS-PLS signature using the DRS had an AUC of 93.9% CI_95%_ (88.4–99.4%) in the 20% test set, which had not been used for discovery (Figure 2), sensitivity of 90.5 CI_95%_ (77.4–97.3) and specificity of 82.4% CI_95%_ (65.5–93.2), with confidence intervals overlapping with the previously identified the 44-transcript elastic net signature for TB/OD (14). The TB/LTBI FS-PLS signature using the DRS had an AUC of 95.4% (CI_95%_ 91.2–99.6%) in the 20% test set, which had not been used for discovery, sensitivity of 91.9 CI_95%_ (78.1–98.3) and specificity of 84.6% CI_95%_ (69.5–94.1), with confidence intervals overlapping with the previously reported 27-transcript elastic net signature (14) (Figure 2, Supplementary Table 5).

**Table 1:** Forward Selection-Partial Least Squares (FS-PLS) signatures for (A) TB/LTBI and (B) TB/OD.

Gene symbol and name^*^	**Illumina Probe ID**	Direction of regulation^*^
**A**		
*GBP6* Guanylate Binding Protein Family Member 6	ILMN_1756953	Up
*TMCC1* Transmembrane and Coiled-Coil Domain Family 1	ILMN_1677963	Down
*PRDM1* PR/SET Domain 1	ILMN_2294784	Up
ARG1 Arginase 1	ILMN_1812281	Down
^*^In patients with TB compared to OD.		
Gene symbol and name^*^	**Illumina Probe ID**	Direction of regulation^*^
**B**		
*FCGR1A* Fc Fragment of IgG Receptor Ia	ILMN_2176063	Up
*ZNF296* Zinc Finger Protein 296	ILMN_1693242	Down
*C1QB* Complement C1q B Chain	ILMN_1796409	Up

* *In patients with TB compared to LTBI*.

*These were taken forward for further characterization and validation using dPCR, and subsequently development as diagnostic biomarkers for TB. Gene names are according to HUGO Gene Nomenclature Committee. LTBI, Latent TB infection; OD, other diseases*.

### Validation of the FS-PLS TB/OD and TB/LTBI Signatures in External Datasets

In order to further validate the performance of the DRS based on the TB/OD four transcript and TB/LTBI three transcript signature, we employed the whole blood expression datasets of Berry et al. (5) and Bloom et al. (28) (GEO: GSE19491, GSE42834) as validation cohorts. The cohorts comprised HIV-uninfected individuals; TB, LTBI, and OD including pneumonia, lung cancer, Still's disease, adult and pediatric Systemic Lupus Erythematosus (ASLE, PSLE), *Staphylococcus*, and *Streptococcus* (Table 2). The TB/OD four transcript signature distinguished TB from all other diseases with an AUC ranging from 88 to 98%, with the exception of sarcoidosis. The TB/LTBI three transcript signature had an AUC of over 91% in the datasets tested.

**Table 2:** Performance of FS-PLS signatures in classifying TB and other diseases (OD), or TB and latent TB infection (LTBI), in other publicly available microarray datasets.

**Comparison**	**Cohort**	***N***	**AUC (95% CI)**
TB vs. OD	Berry (Overall)	213	92.8 (87.6–97.9)
TB vs. OD (non-pulmonary)	Berry (Still's disease)	85	94.7 (88.9–100)
	Berry (adult systemic lupus erythematosus; ASLE)	82	94.6 (89.0–100)
	Berry (pediatric systemic lupus erythematosus; PSLE)	136	91.9 (86.0–97.8)
	Berry (*Staphylococcus*)	94	88.3 (79.6–96.9)
	Berry (*Streptococcus*)	66	98.3 (95.2–100)
TB vs. OD	Bloom (Lung cancer)	51	94.6 (87.2–100)
	Bloom (Pneumonia)	49	90.4 (75.0–100)
	Bloom (Sarcoidosis)	96	63.4 (54.6–74.0)
TB vs. LTBI	Berry (South Africa)	51	98.9 (96.8–100)
	Berry (UK training + test)	72	90.6 (83.2–98.1)

### Clinical Characteristics of Cohorts Used in RT-dPCR Analysis

The clinical characteristics of each disease cohort used for RT-dPCR analysis with the TB/LTBI signature genes and the TB/OD signature genes are shown in Tables 3A,B, respectively. The mean age, body mass index (BMI) and TST induration are shown. The clinical diagnoses of the OD cohort are listed in Supplementary Table 2. The range of diagnoses among this cohort is representative of the variety of conditions that have similar clinical presentations to TB.

**Table 3:** Clinical characteristics of patients used for the RT-dPCR analysis of the (A) TB/OD signature and (B) the TB/LTBI signature.

**Group**	**TB HIV+**	**TB HIV–**	**OD HIV+**	**OD HIV–**
**A**				
Number	10	10	10	10
Age (years) mean (range)	32.3 (26.3–48.0)	31.9 (18.1–60.7)	34.3 (24.9–53.1)	41.1 (19.1–68.1)
Sex (male, %)	50	50	20	30
BMI (kg/m^2^) mean (range)	19.5 (16.8–22.9)	19.4 (15.3–24.2)^a^	24.3 (18.0–39.3)	22.1 (17.2–33.0)^b^
CD4 count (mm^3^) mean (range)	222.5 (29.2–646.0)	NA	252.8 (19.3–838.0)	NA
Anti-retroviral therapy (%)	0	NA	50	NA
^a^One missing value; ^b^Three missing values.				
**Group**	**TB HIV+**	**TB HIV–**	**LTBI HIV+**	**LTBI HIV–**
**B**				
Number	10	10	10	10
Age (years) mean (range)	32.6 (24.2–47.5)	39.7 (290.0–59.8)	36.7 (22.5–53.2)^a^	31.9 (18.9–59.2)
Sex (male, %)	60	50	20	40
BMI (kg/m^2^) mean (range)	20.3 (16.8–25.1)	20.4 (14.3–29.4)	21.6 (16.5–25.8)^a^	21.9 (17.7–29.4)
CD4 count (mm^3^) mean (range)	226.0 (29.2–345.0)	NA	466.1 (227.0–958.0)^b^	NA
Anti-retroviral therapy (%)	0	NA	0	NA
TST induration (mm) mean (range)	ND	ND	22.9 (4.0–50.0)^a^	16.6 (10.0–21.0)

a One missing value;

b *Two missing values*.

*BMI, body mass index; NA, not applicable; ND, not done; TST, tuberculin skin test; LTBI, Latent TB infection; OD, other diseases*.

### Absolute Quantification by RT-dPCR of Genes Comprising the Four-Transcript FS-PLS Signature for TB/OD (Cross-Platform, Cross-Sample Validation)

**Figure 3:**
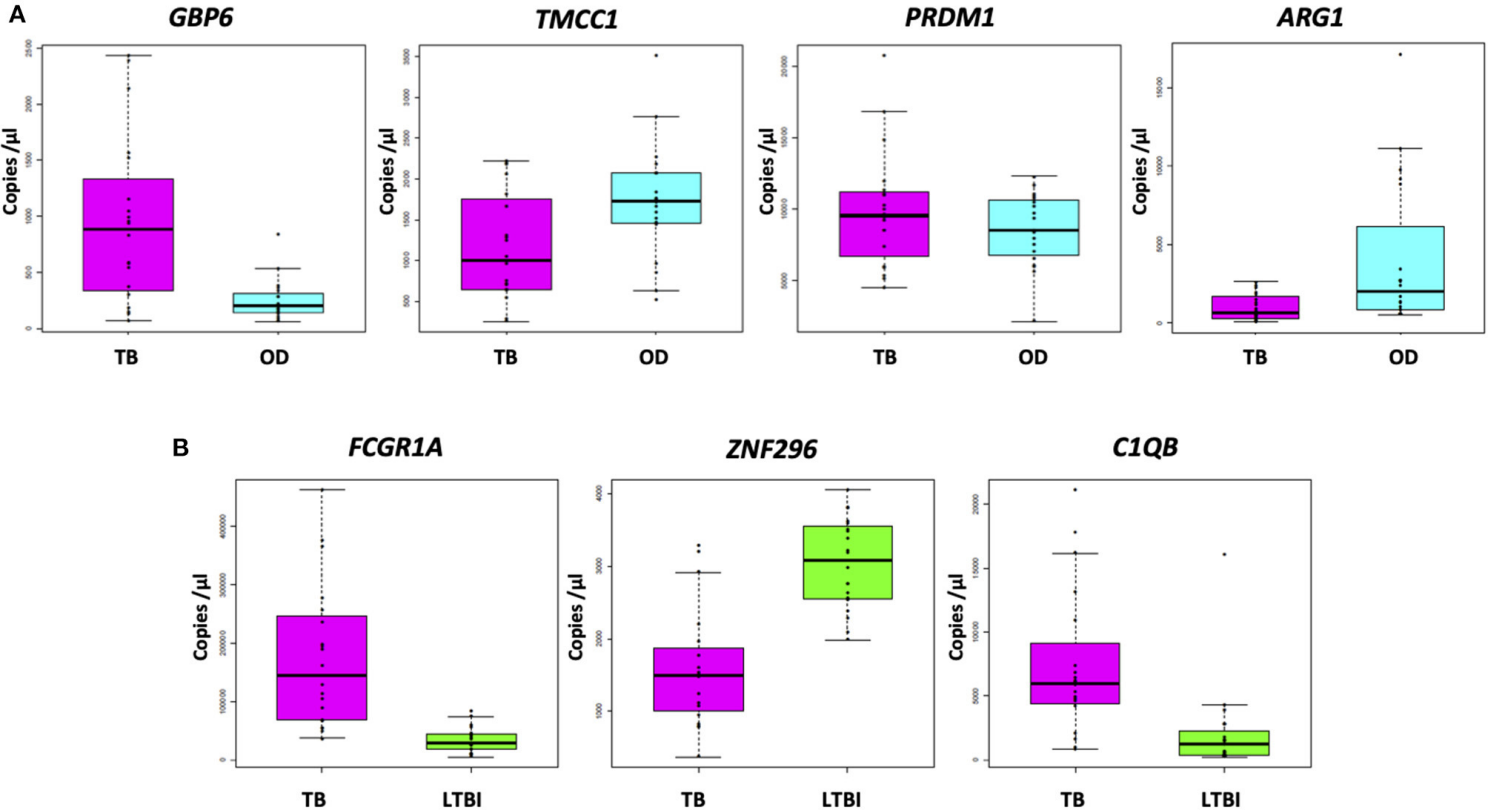
Absolute quantification of signature transcripts, as determined by RT-dPCR, and according to disease group. **(A)** TB/OD signature transcripts. Mann–Whitney *p*: GBP6 3.722 × 10^−4^, TMCC1 2.447 × 10^−2^, PRDM1 4.612 × 10^−1^, and ARG1 2.138 × 10^−3^. **(B)** TB/LTBI signature transcripts. Mann–Whitney *p*: FCGR1A 1.06 × 10^−7^, ZNF296 9.25 × 10^−6^, C1QB 9.65 × 10^−6^. Transcript concentration is expressed as copies per μL. Culture confirmed TB cases are shown in pink (*n*_TB_ = 20), OD cases in cyan (*n*_OD_ = 20), and LTBI individuals in green (*n*_LTBI_ = 20). OD, other diseases; LTBI, latent TB infection.

Figure 3A shows the concentration (in copies per μL) of each of the transcripts comprising the FS-PLS signature for TB/OD in purified RNA from whole blood, as determined by RT-dPCR. *GBP6* transcript levels are higher in TB patients, compared to those with OD. The opposite case is observed for the *ARG1* transcript, which is more abundant in patients with OD compared to TB. For *TMCC1* and *PRDM1*, there is more overlap between concentration values of TB and OD patients. All four of these genes were identified in the 44 gene expression signature for TB/OD, and although GBP6 is induced by the interferon (IFN) cytokine family, its levels were significantly higher in active TB cases when compared to confirmed viral and bacterial infections the GSE73464 (32) and GSE39941 (33) datasets (Supplementary Figure 2). The original concentration (in copies per μL) for the samples stratified by HIV status is shown in Supplementary Figure 4.

### Absolute Quantification by RT-dPCR of Genes Comprising the Three-Transcript FS-PLS Signature for TB/LTBI

The concentrations (in copies per μL) of each of the transcripts comprising the FS-PLS signature for TB/LTBI in purified RNA from whole blood, as determined by RT-dPCR, are shown in Figure 3B. The genes *FCGR1A* and *C1QB* are more abundant in patients with TB compared to LTBI, whereas *ZNF296* is downregulated. All three genes were identified in the original 27 TB/LTBI signature (14). Supplementary Figure 4 shows the concentration (in copies per μL) for the samples stratified by HIV status.

### Correlation of the Microarray Intensity Values and the RT-dPCR Concentration Values

**Figure 4:**
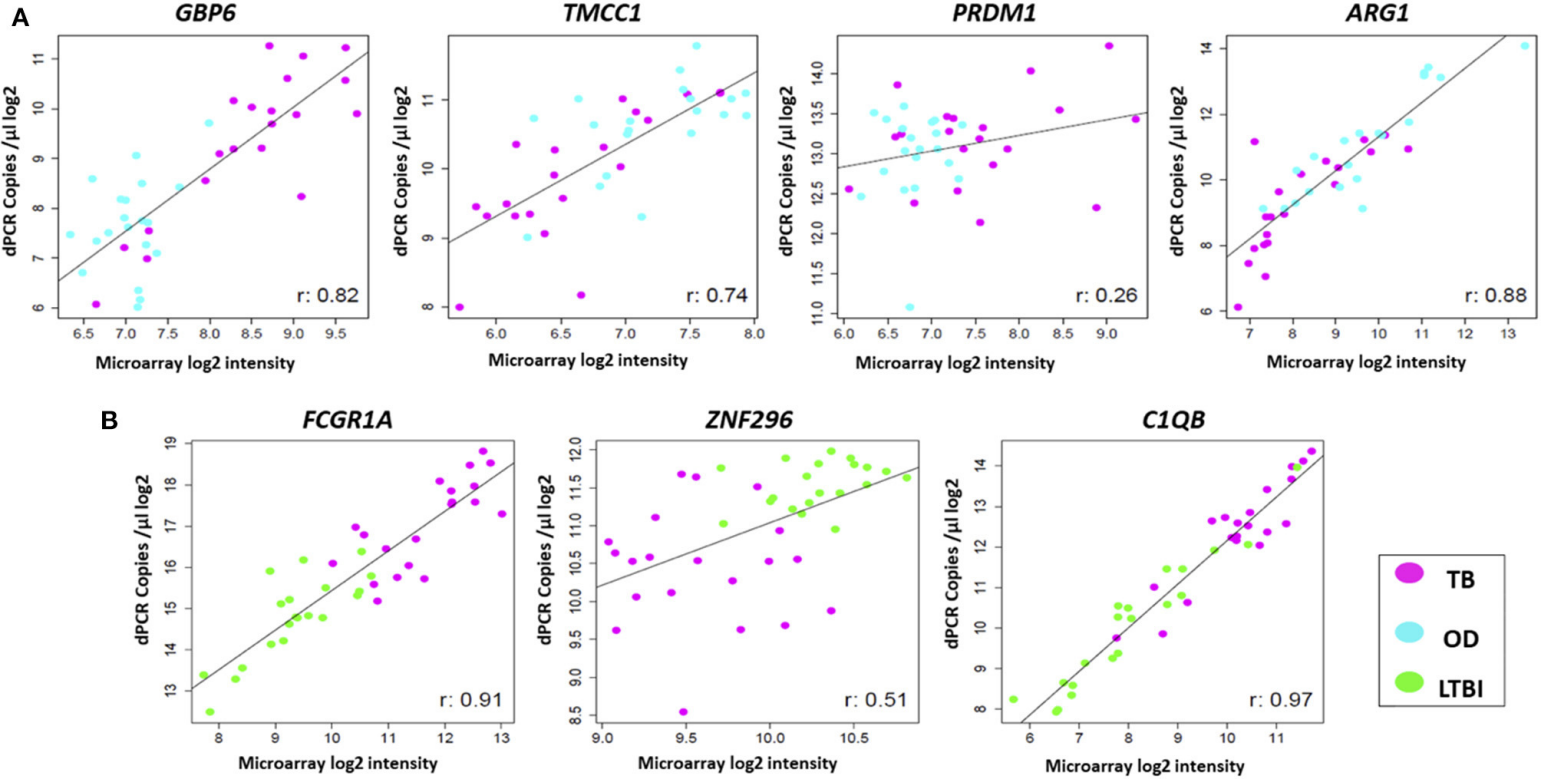
Comparison of expression profiles of common genes and samples between the two platforms. Scatter plots are shown for **(A)** TB/OD signature transcripts and **(B)** TB/LTBI signature transcripts. Plots show log_2_ transformed expression values between two platforms per individual. Pearson's correlation for each transcript is shown. Black line represents the line of best fit. OD, other diseases; LTBI, latent TB infection.

The expression profiles of the seven genes comprising the two signatures described above were compared between the two platforms, at individual sample level. High correlations were observed between the gene expression profiles generated by the two platforms for most of the genes (Figure 4). However, differences in expression profiles were also apparent between the two platforms, with a number of samples/genes exhibiting relatively higher expression values in either platform. Pearson correlation and *p*-values for all the genes can be found in Supplementary Table 6. The Illumina microarray probes and the RT-dPCR TaqMan assays are provided in Supplementary Table 3.

### Performance of the Four-Transcript FS-PLS Signature for TB/OD Using RT-dPCR Analysis Disease Classification in HIV-Infected and HIV-Uninfected Individuals

**Figure 5:**
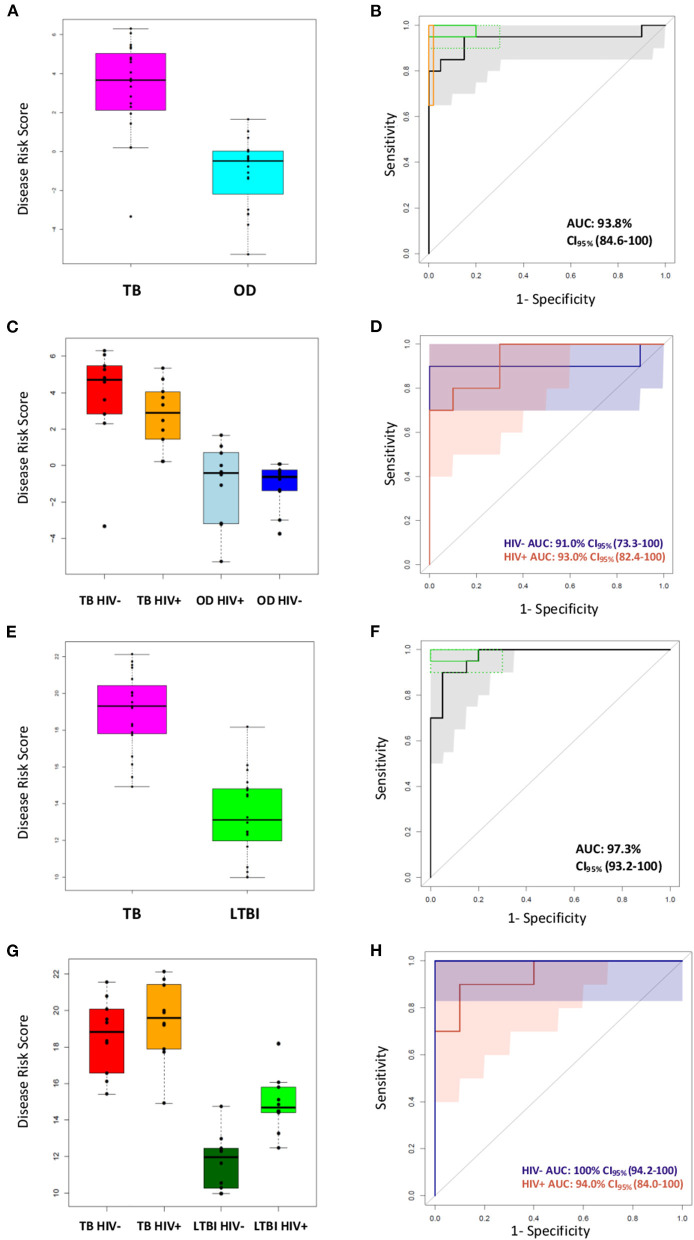
Classification of the SA/Malawi cohorts using the DRS based on the FS-PLS signature RT-dPCR results. **(A)** Box plots of disease risk score (DRS) (TB vs. OD DRS *p* = 1.34 × 10^−7^) and **(B)** Receiver operating characteristic (ROC) curve based on the TB/OD FS-PLS signature applied to the combined TB and OD cohorts (*n*_TB_ = 20, *n*_OD_ =20). Gray shaded areas represent the 95% CIs of the ROC curve sensitivities, plotted at 0.5% specificity intervals. The ROC curve has been benchmarked against target criteria for a tuberculosis triage test: Minimum criteria (90% sensitivity, 70% specificity) are indicated by the dashed green boxes and optimum criteria (95% sensitivity, 80% specificity) are indicated by the green solid boxes; and the minimum criteria for a confirmatory test (65% sensitivity, 98% specificity) which are indicated by the orange box. **(C)** Box plots of DRS (TB vs. OD DRS HIV uninfected *p* = 1.05 × 10^−3^, HIV infected *p* = 4.871 × 10^−4^), and **(D)** ROC curve for TB/OD signature, according to HIV infection status (red line is HIV infected; blue line is HIV uninfected) (*n*_TBHIV−_ = 10, *n*_TBHIV+_ = 10, n _ODHIV−_ = 10, *n*_ODHIV+_ = 10). Blue and orange shaded areas represent the 95% CIs of the ROC curve sensitivities, plotted at 0.5% specificity intervals. **(E)** Box plots of DRS (TB vs. LTBI DRS *p* = 2.83 × 10^−9^) and **(F)** ROC curve based on the TB/LTBI FS-PLS signature applied to the combined TB and LTBI cohorts (*n*_TB_ = 20, *n*_LTBI_ = 20). Gray shaded areas represent the 95% CIs of the ROC curve sensitivities, plotted at 0.5% specificity intervals. The ROC curve has been benchmarked against target criteria for a tuberculosis triage test: Minimum criteria (90% sensitivity, 70% specificity) are indicated by the dashed green boxes and optimum criteria (95% sensitivity, 80% specificity) are indicated by the green solid boxes. **(G)** Box plots of DRS (TB vs. LTBI DRS HIV uninfected *p* = 1.083 × 10^−5^, HIV infected *p*-value 3.248 × 10^−4^, and **(H)** ROC curve for TB/LTBI signature, according to HIV infection status (red line is HIV infected; blue line is HIV uninfected) (*n*_TBHIV−_ = 10, *n*_TBHIV+_ = 10, *n*_LTBIHIV−_= 10, *n*_LTBIHIV+_ =10). 95% confidence intervals are shown in brackets. The orange shaded area represents the 95% CIs of the ROC curve sensitivities, plotted at 0.5% specificity intervals. The blue shaded area for the perfect classifier represents the 95% CI for sensitivity. All *p*-values reported are Mann–Whitney *p*-values. AUC, area under the curve; OD, other diseases; LTBI, latent TB infection.

The performance of the FS-PLS signature for TB/OD was evaluated by applying the DRS to the concentration values that were derived from the RT-dPCR data. Figures 5A–D shows the cross-platform (from microarray to RT-dPCR) and cross-sample (from the training set to the test set) performance of the four gene signature DRS in TB vs. OD. In the combined SA/Malawi HIV-infected and -uninfected cohort, the signature had an AUC of 93.8% (CI_95%_: 82.2–100), a sensitivity of 95.0% (CI_95%_: 85.0–100), and a specificity of 85.0% (CI_95%_: 75.0–100) (Figures 5A,B, Supplementary Table 6). The mean accuracy of classification varied with HIV status, although there was extensive overlap in the 95% confidence intervals. The four gene TB/OD signature had an AUC of 91.0% (CI_95%_: 73.3–100%) among the HIV-uninfected individuals, and an AUC of 93.0% (CI_95%_: 82.4–100%) for the HIV-infected cohort (Figures 5C,D).

### Performance of the Four-Transcript FS-PLS Signature for TB/LTBI Using dPCR Analysis

The performance of the FS-PLS signature for TB/LTBI was evaluated by applying the DRS to the absolute log_2_ transformed concentration values that were derived from the RT-dPCR data. Figures 5E–H show the cross-platform and cross-sample performance of the three gene signature DRS in TB vs. LTBI. In the combined SA/Malawi HIV-infected and uninfected cohort the signature had an AUC of 97.3% (CI_95%_: 93.3–100%), sensitivity of 95.0% (CI_95%_: 85.0–100), and specificity of 85.0% (CI_95%_: 75.0–100) (Figures 5E,F).

As observed previously, the mean accuracy of classification varied with HIV status, although again, there was extensive overlap in the 95% confidence intervals. The four gene TB/LTBI signature had an AUC of 100% (CI_95%_: 94.2–100%) among the HIV-uninfected individuals and an AUC of 94.0% (CI_95%_: 84.1–100%) among HIV-infected cohort (Figures 5G,H, Supplementary Table 6).

### Contribution of Individual Genes to Disease Classification

Finally, we examined the contribution of each gene to the AUC for the classification of the TB/OD and TB/LTBI patients in the microarray and RT-dPCR datasets in a stepwise manner. By definition, in the FS-PLS algorithm, each gene needs to significantly increase the AUC to be included in the signature in the training set (Supplementary Figure 5). The sequential addition of all genes is increasing the AUC in the microarray test and RT-dPCR for the TB/OD comparison, while the inclusion of C1QB in the TB/LTBI signature is not increasing the AUC in the microarray test and RT-dPCR sets, in contrast to the microarray training dataset. As the confidence intervals are largely overlapping, further work is needed to explore the potential of further minimizing the TB/LTBI signature.

## Discussion

In this study, we report a four-gene signature discriminating TB from OD (TB/OD) and a three-gene signature discriminating TB from LTBI (TB/LTBI). These signatures were identified by applying an advanced methodology, FS-PLS, furthering previous work in TB transcriptomics (14, 17). The performance of the two novel transcriptomic signatures, for TB/OD and TB/LTBI was assessed in the 20% test set and publicly available cohorts. The two signatures were subsequently validated using RT-dPCR and samples from the test cohort, confirming their accuracy of patient classification. We also report estimates for the abundance of each of the individual transcripts in the signatures in purified RNA from whole blood. A weighted regression model was not used in this work, reducing the risk of overfitting and providing more flexibility for application transfer in different detection platforms. This work provides compelling evidence of the robustness and reproducibility of the FS-PLS signatures and the DRS in classifying patients with TB, OD, and LTBI and the results presented here support the excellent discriminatory power of both the small gene number TB/OD and TB/LTBI FS-PLS signatures. The point estimates of sensitivity and specificity for our FS-PLS-derived signature, expressed as DRS and measured by both microarray and RT-dPCR, were benchmarked against the WHO TPP recommendations (3). For the microarray test dataset, both the TB/OD and TB/LTBI signatures' point estimates were within the WHO TPP minimum recommendations for a triage test. For the RT-dPCR, the TB/OD signature's point estimates met the WHO TPP requirements of a confirmatory/diagnostic test for TB, and both the TB/OD and TB/LTBI signatures' point estimates overlapped with the requirements of a triage test. While the findings support the discriminatory performance of both signatures, the relatively small sample size and wide confidence intervals of the point estimates should be considered when interpreting these results.

To our knowledge, this study is the first example of the use of RT-dPCR for absolute quantification of transcriptomic signatures in infectious diseases, as anticipated by review articles (34). Previous studies showed that RT-dPCR has a high accuracy for assessing absolute quantification of RNA and did not show significant inter-assay agreement (22). However, it should be noted that the efficiency of reverse transcriptase enzymes can be extremely variable and future investigations will be needed to provide further information on absolute abundances of individual RNA transcripts in purified RNA from whole blood. Nevertheless, the concentration values reported in this study provide novel insights that could be of significant use to the diagnostics development research community, providing information regarding the required limits of detection and dynamic range for assays designed to detect signature transcripts. Although high correlation was observed between the gene/sample measurements for the two platforms for most of the genes, the differences reported highlight that a larger number of highly correlated candidate biomarker genes and different target regions within the genes themselves need to be screened with technology reflective of the point-of-care platforms intended to be used in order to ensure maximum diagnostic potential.

Clinical applications of dPCR exploit its ability to perform absolute quantification of nucleic acids without the need for rigorous calibration or standardization between laboratories. This advantage is a result of the design of dPCR assays, which involve large numbers of reaction partitions, and the Poisson statistics that are used to calculate initial concentrations of nucleic acids (21). RT-dPCR and dPCR have been used to determine copy numbers for a range of pathogens, including the hepatitis B virus, HIV, *Mycobacterium tuberculosis, Helicobacter pylori*, and *Plasmodium* spp. (23). While dPCR is more technologically advanced than qPCR, offering absolute rather than relative quantification, the implementation of dPCR in clinical laboratories has been impeded by its relatively low throughput, higher complexity and cost. However, as new instrumentation for dPCR becomes more widely available and simpler to use, it is highly likely that it will play a key role in diagnostic laboratories in the near future (23).

Out of the four transcripts in the TB/OD transcript signature *GBP6* and *PRDM1* are upregulated, and *TMCC1* and *ARG1* are downregulated, in patients with TB compared to OD. Genes in the guanylate-binding protein gene cluster (such as *GBP2, GBP5*, and *GBP6*) appear in numerous TB gene signatures (10). These are induced by the interferon (IFN) cytokine family (35) and have been shown to be important for cell-autonomous defense against intracellular pathogens (36). *PRDM1* encodes a DNA-binding protein that acts as a transcriptional repressor of various genes, including IFN-β (37) by binding specifically to the PRDI (positive regulatory domain I element). *PRDM1* has also been shown to regulate the differentiation of B cells into plasma cells that produce antibodies, as well as myeloid cells, such as macrophages and monocytes (38). Little is known about the function of *TMCC1* in TB pathogenesis, but expression of *ARG1* is induced by toll-like receptor signaling in macrophages (39). The gene product, ARG1, plays an important role in the production of nitric oxide (NO), used to kill intracellular pathogens, when nitric oxide synthase-2 (NOS2) is unable to metabolize arginine in hypoxic environments, such as the granuloma (40). ARG1 is able to produce NO in the absence of oxygen and is therefore critical for the control of intracellular TB (41).

The three gene signature for TB/LTBI reported here consists of two genes that are upregulated (*FCGR1A* and *C1QB*) and one gene that is downregulated (*ZNF296*) in TB compared to LTBI. *FCGR1A* appears in a number of other gene expression signatures for TB (10), and was the most discriminatory gene in a three-gene signature for TB/LTBI (6). Fc receptors (FcR) play an important role in regulating the immune system and are expressed by a number of innate immune effector cells, particularly monocytes, macrophages, dendritic cells, basophils and mast cells (42). It has been shown that the monocytic THP-1 cell line upregulates surface expression of Fcγ-RI in response to IFN-γ (43). *C1QB* encodes a component of the complement 1 (C1Q) complex, part of the complement immune system. Expression of genes encoding components of C1Q have been shown to correlate with the progression of active TB compared to HC and LTBI cohorts (44) and a recent study showed that, in four independent cohorts, components of the C1Q complex are elevated in patients with active TB compared to those with LTBI (45). *ZNF296* encodes a member of the C2H2 zinc-finger protein family, which contain DNA binding motifs often found in transcription factors. A microarray study identified this gene as upregulated in response to viral infection (46). The TB/LTBI signature presented here was evaluated by Gupta et al. (8) for the purposes of predicting progression from LTBI to active TB disease. Out of a total of 17 candidate signatures identified, eight accurately predicted incipient TB among people at risk of disease over a two-year time period with AUCs ranging from 70% (CI_95%_: 64–76%) to 77% (CI_95%_: 71–82%). Our TB/LTBI signature ranked second in terms of point estimate for AUC, with overlapping 95% confidence intervals with the other top-ranking signatures. Significantly lower AUCs were found for the remaining nine signatures.

This study has certain limitations. Although a case-control validation study is an important step in the biomarker discovery pipeline, it has certain limitations in extrapolating how the findings would transfer in a real-world clinical setting. A prospective cohort study design where positive and negative predictive values of a test would be the next step to evaluate the signatures' potential and applicability. This study is further limited by the small sample size used for the RT-dPCR evaluation, which is reflected in the relatively wide 95% confidence intervals reported for the classification measure.

It is widely accepted that TB diagnosis using transcriptomic signatures offers a number of clear advantages over various sputum-based techniques. However, there are a number of technical challenges of detecting mRNA from whole blood, including sample processing to extract mRNA transcripts that is generally intracellular and inherently less stable than DNA, and that can vary in concentrations by multiple orders of magnitude between samples.

The gene expression signatures for TB/LTBI and TB/OD reported in this study represent extremely promising biomarkers for TB, particularly since they can be measured in whole blood and comprise few analytes. A number of technologies exist that might facilitate their translation into a test, which could include the use of nanomaterials, to quantify mRNA transcripts without an amplification step (47). A whole blood-based diagnostic test for TB would transform the diagnostic pipeline and enable earlier treatment commencement for patients that would otherwise be missed, and thus prevent onward transmission of the disease, contributing toward paving the way for the end of the TB epidemic by 2030, Goal 3.3 of the Sustainable Development Goals, as set out by the United Nations (48).

## Data Availability Statement

The datasets presented in this study can be found in online repositories. The names of the repository/repositories and accession number(s) can be found at: www.ncbi.nlm.nih.gov/geo/, GSE37250.

## Ethics Statement

The studies involving human participants were reviewed and approved by Human Research Ethics Committee of the University of Cape Town, South Africa (HREC012/2007) The National Health Sciences Research Committee, Malawi (NHSRC/447) The Ethics Committee of the London School of Hygiene and Tropical Medicine (5212). The patients/participants provided their written informed consent to participate in this study.

## Author Contributions

HG, MK, NF, HD, LC, RW, and ML: data curation. HG, MK, DH-C, CZ, and LC: formal analysis (application of statistical, mathematical, computational, or other formal techniques to analyze or synthesize study data) and validation (verification, whether as a part of the activity or separate, of the overall replication/reproducibility of results/experiments and other research outputs). MK, VW, SA, AC, BE, FK, PL, THMO, MH, NF, HD, LC, RW, and ML: funding acquisition. HG and MK: investigation (conducting a research and investigation process, specifically performing the experiments, or data/evidence collection). HG, MK, MA, CZ, and LC: methodology. HG, MK, VW, RW, and ML: project administration. HG, MK, MA, AC, FK, PL, THMO, MH, NF, HD, LC, RW, and ML: resources (provision of study materials, reagents, materials, patients, laboratory samples, animals, instrumentation, computing resources, etc.). MK, DH-C, CZ, and LC: software programming, software development, designing computer programs, implementation of the computer code and supporting algorithms, testing of existing code components. MK, MH, NF, HD, LC, RW, and ML: supervision. HG and MK: visualization (preparation, creation and/or presentation of the published work, specifically visualization/data presentation). HG, MK, and ML: writing—original draft preparation. All authors: conceptualization of study, writing—review and editing. All authors contributed to the article and approved the submitted version.

## Conflict of Interest

The authors declare that the research was conducted in the absence of any commercial or financial relationships that could be construed as a potential conflict of interest.
